# Roles of Circular RNAs in Neurologic Disease

**DOI:** 10.3389/fnmol.2016.00025

**Published:** 2016-04-13

**Authors:** Yiye Shao, Yinghui Chen

**Affiliations:** ^1^Department of Neurology, Jinshan Hospital, Fudan UniversityShanghai, China; ^2^Department of Neurology, Shanghai Medical College, Fudan UniversityShanghai, China

**Keywords:** circular RNA, non-coding RNA, neurological disease, microRNA, pathogenesis

## Abstract

Circular RNAs (circRNAs) are a novel type of endogenous noncoding RNA receiving increasing attention. They have been shown to act as a natural microRNA sponges that repress the activity of corresponding miRNAs by binding with them, thus regulating target genes. Numerous studies have shown that miRNAs are involved in the pathogenesis of neurological diseases. Therefore, circRNAs may act as important regulatory factors in the occurrence and development processes of neurological disease.

Differences between species are embodied in both gene numbers and the regulation of gene expression. Recent studies have demonstrated that non-coding RNAs play an important role in regulating gene expression. CircRNA was first discovered as a novel type of endogenous non-coding RNAs in the 1970s (Sanger et al., 1976; Arnberg et al., 1980; Cocquerelle et al., 1993), however, the function of these special structures has only recently been revealed and appreciated. In this review, we will highlight recent studies of the latest progress in the circRNAs field, in the context of neurological diseases.

## CircRNA structure

**Figure 1:**
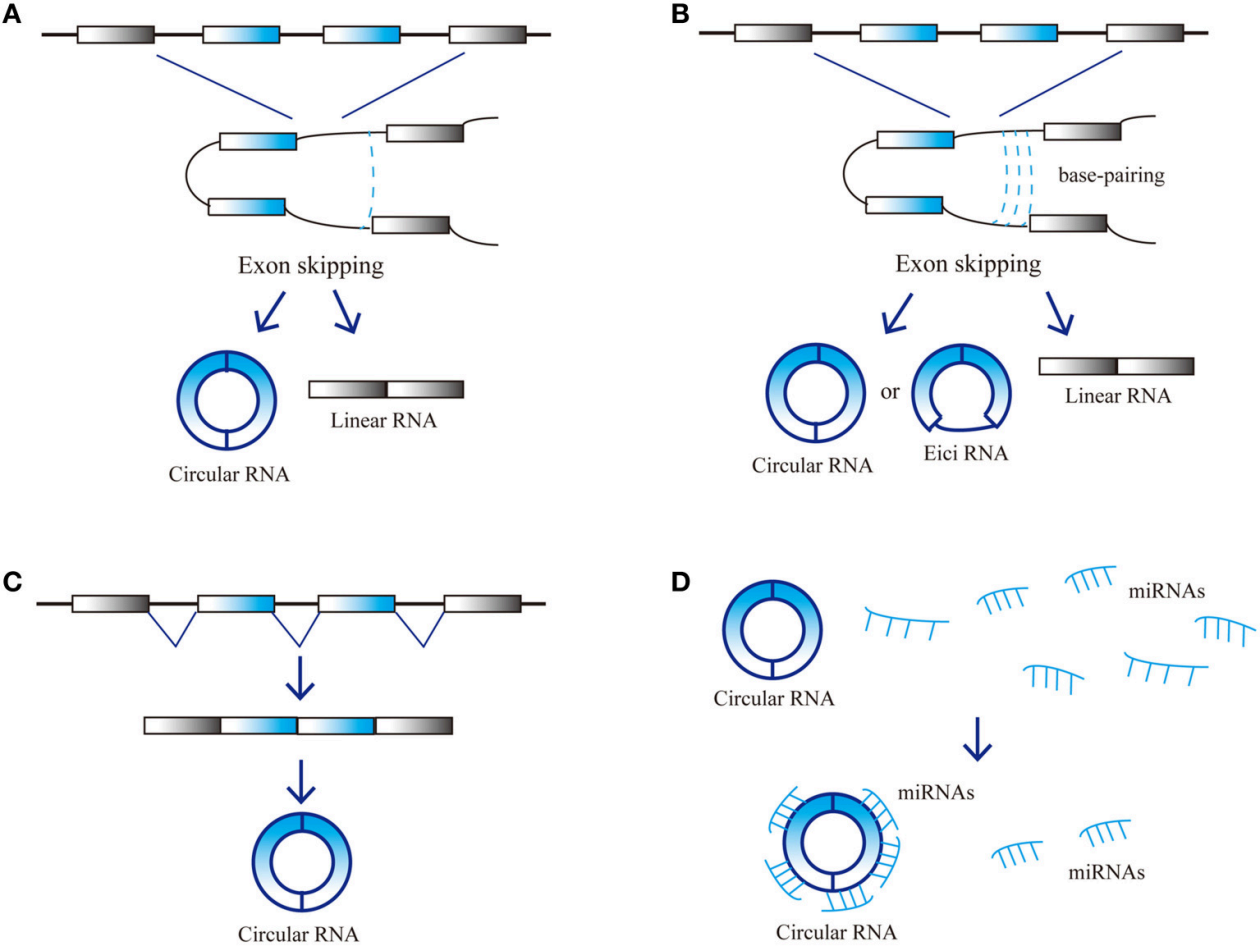
**Possible models and function of circRNA biogenesis. (A)** Lariat-driven circularization, **(B)** Intron-pairing-driven circularization, **(C)** Resplicing-driven circularization, and **(D)** Function as miRNAs sponge.

CircRNAs are a novel type of endogenous noncoding RNA, different from the linear RNAs, which form closed loop structures with a covalent bond linking the 3′ and 5′ ends. Consequently, they do not possess polyadenylated tails (Jeck et al., 2013; Chen et al., 2015). Classic RNA detection methods can only separate RNA molecules with polyadenylated tails, so circRNAs were ignored in previous studies. Since the development of high-throughput sequencing techniques and bioinformatics analysis programs, researchers have discovered thousands of circRNAs in mammalian cells (Salzman et al., 2012; Memczak et al., 2013). CircRNAs are abundant in plasma, sometimes at levels 10-folds higher than the corresponding linear mRNAs, due to their increased stability. Nuclease hydrolyzes can recognize the tails of linear RNAs, while circular RNAs form covalently closed loop structures that protect them. As a result, circular RNAs are not susceptible to degradation by RNA exonuclease or RNase (Salzman et al., 2012; Jeck et al., 2013; Memczak et al., 2013). CircRNAs are highly enriched in eukaryotic organisms and display elevated sequence conservation with specific expression in various tissues during different developmental stages. CircRNAs mainly arise from the exons of protein-coding genes and can also be derived from intronic areas, untranslated regions (UTRs), intergenic loci and antisense sequences of known transcripts (Jeck et al., 2013; Memczak et al., 2013). CircRNA biogenesis remains unclear, several models were proposed to explain the possible formation of ecircRNAs: Lariat, Intron-pairing, and resplicing-driven circularization (Figure 1; Chen et al., 2015). Some circRNAs may have miRNA response elements (MREs) and can interact with miRNAs (Hansen et al., 2011).

## CircRNA function

Due to their high expression level and stability, circRNAs can act as competitive endogenous RNAs (ceRNAs). CeRNAs contain shared MREs and can compete for miRNA binding. Furthermore, circRNAs have the same selective capture of transcribed sequences with corresponding linear mRNAs, which may perform specific functions through influencing the combination of other RNAs. CircRNAs play an important role in the micro-adjustment of miRNA expression levels. CircRNAs can perturb miRNA function by competing with miRNA combination, thus preventing miRNA's effect on the posttranslational repression of target-coding RNA species, and then regulating the expression levels of target genes (Hansen et al., 2013a; Rybak-Wolf et al., 2015). Studies have revealed that the ability of circRNAs to combine with miRNAs is 10 times higher than other known transcriptions (Chen et al., 2015). A circRNA antisense to the cerebellar degeneration-related protein 1 transcript (CDR1as) can harbor more than 70 miR-7 binding sites and cannot be degraded by RNA-induced silencing complexes (RISCs) (Hansen et al., 2013a). Acting as a natural miRNA sponge, CDR1as can negatively regulate miR-7 (Salmena et al., 2011; Danan et al., 2012; Memczak et al., 2013). Therefore, CDR1as is also called the circular RNA sponge for miR-7 (CiRS-7). High CDR1as expression can decrease miR-7 activity by binding to it and thus increasing target gene expression; therefore, low CDR1as expression decreases miR-7 target gene expression. Moreover, CDR1as seems to be degraded by miR-671 but not miR-7 (Hansen et al., 2011, 2013a; Memczak et al., 2013). This indicates that circRNAs can transport miRNAs. CDR1as may first combine with miR-7 and transport it to specific sites, where miR-671 can degrade it and release miR-7. This characteristic is bound to affect ceRNA function because the combination and release of multiple miRNAs by circRNAs can affect hundreds of transcripts (Taulli et al., 2013). Studies have demonstrated that circRNA from sex-determining region Y (sry) harbors 16 miR-138 binding sites and can act as natural miRNA sponges to repress miR-138 activity (Hansen et al., 2013a). There are several other possible circRNA functions. First, they can regulate the expression of other RNAs through partial base pairing. For instance, CDR1as can bind with CDR1 mRNA based on complementary base pairing, thus increasing CDR1 mRNA stability (Hansen et al., 2011). Second, circRNA can combine with proteins and regulate their activity or recruit the components of multiprotein complexes (Bohjanen et al., 1996; Hansen et al., 2013a; Hentze and Preiss, 2013; Memczak et al., 2013). For example, CDR1as can bind with Argonaute (AGO) protein. Finally, circRNAs can act as translation templates to encode proteins (Chen and Sarnow, 1995; Perriman and Ares, 1998). CircRNAs may play significant roles in nervous system. Studies have found that CircRNAs are highly abundant in central nervous system and are differentially expressed in the brain. circRNAs are highly enriched in synapses and the expression level of circRNAs are differentially during neuronal differentiation (Rybak-Wolf et al., 2015). The above characteristics suggest that circRNAs may participate in development of nervous system, especially in synapse development and plasticity.

## CircRNA in neurological disease

Recent investigations have suggested that circRNAs may play crucial roles in the occurrence and development of neurological diseases and therefore have potential as novel biomarkers (Table 1). At present, few studies have directly assessed the functions and mechanisms of circRNAs in neurological diseases; rather, most are only related to circRNAs functions as miRNA regulators. One publication reported that circRNAs are extraordinarily enriched in the brain, especially in neuropils and dendrites, and may participate in regulating synaptic function and neural plasticity (You et al., 2015). These characteristics suggest that circRNAs could play important roles in nervous system diseases such as epilepsy, Parkinson's disease (PD), and Alzheimer's disease (AD). CDR1as overexpression in zebrafish embryos decreased midbrain size, which is the same effect observed following miR-7 silencing (Memczak et al., 2013). A large number of studies have demonstrated that miR-7 influences different signaling pathways by acting on other regulatory factors such as epidermal growth factor receptor (EGFR), insulin receptor substrate-1 (IRS-1), IRS-2, p21-activated kinase-1 (Pak1), and Raf1 (Reddy et al., 2008; Webster et al., 2009). Therefore, CDR1as may be an important regulatory factor in the pathogenesis and progression of neurological diseases.

**Table 1:** **Functional mechanism of Circular RNAs in neurological disease**.

**Neurological disease**	**Circular RNA**	**Targets**	**Mechanisms**
AD	CDR1as	miR-7	miR-7 can down-regulate AD-relevant targets, such as UBE2A, which play an important role in the clearance of amyloid peptides in AD (Bingol and Sheng, 2011; Lukiw, 2013)
	Sry	miR-138	miR-138 participate in learning and memory ability by regulating APT1 (Hansen et al., 2013a)
PD	CDR1as	miR-7	miR-7 can downregulate α-synuclein expression and protect cells against oxidative stress (Junn et al., 2009)
Cerebrovascular disease	cANRIL	INK4a	cANRIL can influence INK4/ARF expression and increase the risk of ASVD (Salzman et al., 2013)
Inflammatory neuropathy	hsa-circRNA 2149		Specific expression in leukocytes
	CircRNA100783		CircRNA100783 may be involved in chronic CD28-associated CD8(+)T cell aging (Wang et al., 2015)
	Sry	miR-138	miR-138 can balance the expression between Th1 and Th2 through suppressing the function of RUNX3 (Fu et al., 2015)
Nervous System Neoplasms	CDR1as	miR-7	miR-7 can repress the expression of EGFR, IRS-1 and IRS-2 thus reduce the active and aggressive of glioblastoma (Liu et al., 2014)
Prion disease	CDR1as		PrPC can up-regulate expression of CDR1as (Satoh and Yamamura, 2004; Satoh et al., 2009)

## CircRNA and AD

Functional deficiency of CDR1as can upregulate miR-7 expression and may lead to the downregulation of AD-relevant targets, such as ubiquitin protein ligase A (UBE2A; Bingol and Sheng, 2011; Lonskaya et al., 2013; Lukiw, 2013). This autophagic protein is important for clearing amyloid peptides and is depleted in the AD brain (Lonskaya et al., 2013). This suggests that CDR1as may participate in AD pathogenesis. In addition, circRNA from sry can act as a natural miRNA sponge to repress miR-138 activity (Hansen et al., 2013a). Some studies have shown miR-138 influences learning and memory abilities by regulating acyl protein thioesterase 1 (APT1; Tatro et al., 2013; Schröder et al., 2014).

## CircRNA and PD

As mentioned previously, CDR1as is a negative regulator of miR-7 (Salmena et al., 2011; Danan et al., 2012; Memczak et al., 2013). Investigations have revealed that miR-7 can downregulate the expression of α-synuclein, which is the main component of Lewy bodies in the PD brain. High neuronal expression of α-synuclein protein is highly implicated in PD pathogenesis. Moreover, miR-7-induced α-synuclein downregulation can also protect cells against oxidative stress (Junn et al., 2009). miRNA-7 can also protect against 1-methyl-4-phenylpyridinium-induced cell death by targeting the nuclear factor (NF)-κB signaling pathway (Choi et al., 2014).

## CircRNA and cerebrovascular disease

Circular antisense non-coding RNA in the INK4 locus (cANRIL) is an antisense transcript from the cyclin-dependent kinase 4 inhibitor (CDK4a, also called INK4a) alternative reading frame locus (Salzman et al., 2013). Single nucleotide polymorphisms (SNPs) near INK4/ARF are associated with atherosclerotic vascular disease. The cANRIL locus is within the SNPs and the nearest coding gene, and cANRIL can influence INK4/ARF expression and increase the risk of ASVD (Burd et al., 2010). This observation indicates that circRNA may be involved in the development of atherosclerotic cerebrovascular disease.

## CircRNA and inflammatory neuropathy

Some circRNAs seem to have virus miRNA binding sites and therefore can affect immune responses. For example, hsa-circRNA 2149 contains 13 unique, head-to-tail spanning reads. Researchers detected hsa-circRNA 2149 in CD19+ leukocytes but not CD341 leukocytes, neutrophils, or HEK293 cells. Besides, circular RNA100783 may be involved in chronic CD28-associated CD8(+)T cell aging and could therefore be a novel biomarker for this conditions (Wang et al., 2015). CircRNA from sry can repress miR-138 activity. Studies found that miR-138 could balance T helper 1 (Th1) and T helper 2 (Th2) expressions through suppressing the function of runt-related transcription factor 3 (RUNX3; Fu et al., 2015). The above studies suggest that circRNA may participate in inflammatory reactions that induce neuropathy.

## CircRNA and nervous system neoplasms

Several circRNAs play critical roles in cancer related biological processes and are dysregulated in cancer tissues (Li et al., 2015a,b; Qin et al., 2016). CDR1as was first found in brain tissue and is widely expressed in the nervous system, especially the cerebrum. Expression analyses of various tumor cell lines showed widespread expression of CDR1as in neuroblastomas and astrocytoma (Dropcho et al., 1987; Chen et al., 1990; Hansen et al., 2013b). Researchers measured miRNA expressions in different tumor cell lines and found that compared to normal brain tissue, miR-7 was down-regulated in astrocytoma and neuroblastoma, Research on a glioblastoma cell line demonstrated that miR-7 could repress EGFR expression and also downregulate IRS-1 and IRS-2 expression by suppressing the activity of protein kinase B (PKB; Liu et al., 2014). CDR1as acts as negative regulator of miR-7 (Hansen et al., 2013a). These lines of evidence indicate that circRNAs may be involved in the pathogenesis and progression of nervous system neoplasms.

## CircRNA and prion diseases

Prion diseases are devastating neurodegenerative disorders including Creutzfeldt–Jakob disease (CJD), kuru, Gerstmann-Straussler syndrome, and fatal familial insomnia (FFI; Prusiner, 1998). Most prion diseases are infectious via transmissible particles composed of scrapie prion protein (PrPSc), an isomer of cellular prion protein (PrPC). Studies have revealed that CDR1as expression is induced by PrPC overexpression (Satoh and Yamamura, 2004; Satoh et al., 2009). Therefore, CDR1as may be involved in the prion disease pathogenesis.

## Conclusions

CircRNA function and their relationships with neurological diseases remain to be fully elucidated, and some problems remainto be solved. Due to the abundance and stability of circRNAs *in vivo*, they may be useful clinical diagnosis biomarkers in the future. Meanwhile, the regulatory effects of circRNAs on genes may be considered as treatment targets. In addition, the ability of circRNAs to act as sponges of related miRNAs can be exploited as a novel technology to achieve gene regulation, different cytotypes could differentially be regulated with a selective silencing approach. Further study on circRNA structure and function will improve our understanding regarding neurological disease pathogenesis and lead to new diagnostic and treatment methods.

## Author contributions

YS conceived the content and wrote the critical review. YC provided the ideas and supervised the work. Both authors read and approved the final version of the manuscript.

### Conflict of interest statement

The authors declare that the research was conducted in the absence of any commercial or financial relationships that could be construed as a potential conflict of interest.
